# Treating the dysfunctional placenta

**DOI:** 10.1530/JOE-17-0185

**Published:** 2017-05-03

**Authors:** Colin P Sibley

**Affiliations:** 1 Maternal and Fetal Health Research CentreDivision of Developmental Biology and Medicine, School of Medical Sciences, Faculty of Biology, Medicine and Health, University of Manchester, Manchester, UK; 2 St Mary’s HospitalCentral Manchester University Hospitals NHS Foundation Trust, Manchester Academic Health Science Centre, Manchester, UK

**Keywords:** placenta, dysfunction, treatment, pre-eclampsia, fetal growth restriction

## Abstract

Placental dysfunction underlies major obstetric diseases such as pre-eclampsia and fetal growth restriction (FGR). Whilst there has been a little progress in prophylaxis, there are still no treatments for placental dysfunction in normal obstetric practice. However, a combination of increasingly well-described *in vitro* systems for studying the human placenta, together with the availability of more appropriate animal models of pre-eclampsia and FGR, has facilitated a recent surge in work aimed at repurposing drugs and therapies, developed for other conditions, as treatments for placental dysfunction. This review: (1) highlights potential candidate drug targets in the placenta – effectors of improved uteroplacental blood flow, anti-oxidants, heme oxygenase induction, inhibition of HIF, induction of cholesterol synthesis pathways, increasing insulin-like growth factor II availability; (2) proposes an experimental pathway for taking a potential drug or treatment for placental dysfunction from concept through to early phase clinical trials, utilizing techniques for studying the human placenta *in vitro* and small animal models, particularly the mouse, for *in vivo* studies; (3) describes the data underpinning sildenafil citrate and adenovirus expressing vascular endothelial growth as potential treatments for placental dysfunction and summarizes recent research on other potential treatments. The importance of sharing information from such studies even when no effect is found, or there is an adverse outcome, is highlighted. Finally, the use of adenoviral vectors or nanoparticle carriers coated with homing peptides to selectively target drugs to the placenta is highlighted: such delivery systems could improve efficacy and reduce the side effects of treating the dysfunctional placenta.

## Introduction

By the end of pregnancy the human placenta is the largest endocrine organ in a woman, secreting large quantities of an array of hormones into both maternal and fetal plasma (Costa 2016). These hormones control maternal adaptation to pregnancy, the timing of parturition, matching of supply with demand for nutrients by the fetus to enable normal growth (Sferruzzi-Perri & Camm 2016) and indeed control of placental development itself. It is therefore not surprising that defective placentation and the consequently dysfunctional placenta is a direct cause of what has been termed the ‘Great Obstetrical Syndromes’: pre-eclampsia, fetal growth restriction (FGR), preterm labour, preterm premature rupture of membranes, late spontaneous abortion and abruption placentae (Brosens *et al*. 2011). This involvement of the placenta in pregnancy complications now seems self-evident, and is indeed inherent in the old-fashioned obstetric phrase ‘placental insufficiency’. However, the focus of obstetric intervention for these conditions has been managing the outcomes (e.g. delaying labour or inducing labour). In fact, there have been very few therapeutic advances in obstetrics in the last 30 years (Fisk & Atun 2008, Chappell & David 2016). Whilst aspirin prophylaxis for women at high-risk pre-eclampsia, for example, is now a recommendation (https://www.nice.org.uk/guidance/qs35/chapter/quality-statement-2-antenatal-assessment-of-pre-eclampsia-risk), it is only recently that researchers have considered the possibility of treating the dysfunctional placenta itself. This review focuses on treating the dysfunctional placenta in pre-eclampsia and FGR. It will consider: the pathophysiology of these pregnancy complications and candidate drug targets; why there are currently no treatments for placental dysfunction in general obstetric practice and pre-clinical strategies and models for developing such treatments; potential treatments for placental dysfunction arising from recent research; and finally, future opportunities and challenges to this emerging field of research will be considered.

## Pathophysiology of pre-eclampsia and FGR and therapeutic targets

The signs of pre-eclampsia are maternal hypertension with endothelial and renal dysfunction; in extreme cases, or if not treated, pre-eclampsia may progress to eclampsia with cerebral oedema, epileptic fits and death (Duley 2009). Pre-eclampsia remains the major cause of death of pregnant women worldwide with over 50,000 deaths per annum (Duley 2009). FGR is defined as a failure of the fetus to reach its genetic growth potential; its diagnosis is complicated by the difficulty in separating the normal small fetus from the truly growth-restricted one (Baschat & Galan 2017). Although beyond the scope of this review, it seems likely that diagnosis of placental dysfunction will eventually be a better diagnostic indicator of FGR than is fetal size or growth trajectory (Benton *et al*. 2016). FGR is strongly associated with stillbirth and neurodevelopmental disorders in the survivors (Flenady *et al*. 2011, Baschat & Galan 2017). Around 50% of stillbirths are associated with FGR, and such deaths still occur in 1/200 pregnancies with massive socio-economic consequences (Flenady *et al*. 2011, Heazell *et al*. 2016). FGR and pre-eclampsia frequently occur together.

The origins of the placental dysfunction underlying pre-eclampsia and FGR (as well as the other Great Obstetric Syndromes) are predominantly in abnormal physiological conversion of the spiral arteries in the endometrium supplying blood to the intervillous (maternal) blood space (IVS) of the placenta (Brosens *et al*. 2011). In normal pregnancy, following the formation of the placenta extravillous cytotrophoblast (EVT) cells invade the spiral arteries so that both the endothelium and smooth muscle of the vessels are eroded (Burton *et al*. 2017). This invasion by the EVT is extensive and initially plugs the terminal end of the vessels, preventing any blood flow into the intervillous space until about week 10 when the plug becomes disrupted. The invasion also results in the distal end of the spiral arteries becoming converted from small calibre vessels, with normal vasoreactivity, into wide conduits. The proximal ends of the spiral arteries are not converted by EVT but do dilate during early pregnancy, as do other parts of the uterine vasculature, most likely under the influence of oestrogen. The consequent fall in resistance enables an increase in blood flow to the uterus from about 45 mL/min pre-pregnancy to 750 mL/min during pregnancy. At the same time, the dilation of the terminal ends of the spiral arteries probably ensures that blood flow into the IVS is relatively slow, and non-turbulent, helping to prevent damage to the villous tree and facilitating diffusional exchange across the placenta.

**Figure 1:**
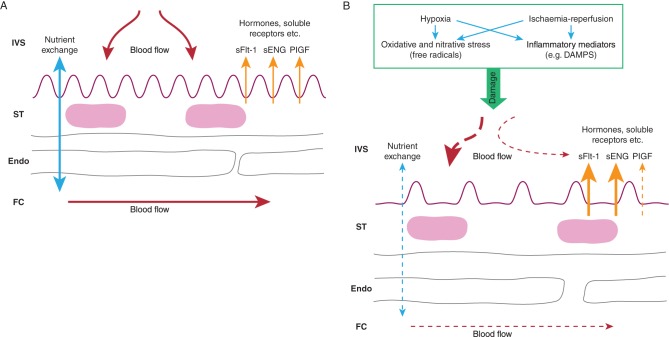
(A) Image showing the main structural and functional elements of the exchange barrier of the normal placenta: IVS intervillous space with maternal blood; ST syncytiotrophoblast – the placental epithelium; Endo fetal capillary endothelium; FC fetal capillary with fetal blood. (B) Consequences of abnormal spiral artery invasion and conversion on the structure and function of the placenta. Hypoxia and ischaemia-reperfusion may lead to the formation of free radicals and/or inflammatory mediators such as damage-associated molecular patterns (DAMPS). These could cause: abnormal blood flow patterns in the IVS; decreased fetoplacental blood flow; altered structure of the exchange barrier (e.g. reduced surface area and increased thickness of the ST) with reduced nutrient transfer; increases and decreases in secretion of hormones, soluble receptors and other placental proteins and factors. All of these are potential targets for treatments of placental dysfunction.

Pre-eclampsia and FGR are associated with failure in EVT invasion and consequent malformation of the maternal circulation of the placenta (Brosens *et al*. 2011, Burton *et al*. 2017). Physiological conversion is restricted to the superficial endometrial parts of the spiral arteries or does not happen at all. Consequently: (i) with no distal dilation, maternal blood will enter the IVS at greater speed disrupting the villous tree, with formation of intervillous blood lakes, thrombosis and fibrin, which will affect nutrient exchange processes; (ii) the spiral arteries will maintain a greater than normal ability to vasodilate and vasoconstrict, causing massive oxidative and nitrative stress in the placenta through ischaemia-reperfusion processes and (iii) there may be atherosclerotic changes in the distal ends of the arteries further altering blood flow to the IVS. These abnormalities in spiral artery re-modelling therefore lead to the poor uteroplacental blood flow, which compromises the formation of the fetoplacental circulation and villous tree of the placenta with the consequent dysfunction underlying pre-eclampsia and FGR (Fig. 1). In pre-eclampsia, it is proposed that hypoxia and/or ischaemia-reperfusion lead to the release of free radicals and inflammatory mediators causing cellular stress in the syncytiotrophoblast (the placental epithelium) (Powe *et al*. 2011, Burton *et al*. 2017). This leads to the release of abnormal levels of factors into the maternal circulation, which causes an exaggerated inflammatory response and defective proliferation and survival of endothelial cells resulting in endothelial and, more specifically, renal dysfunction. These placental factors include excess release of soluble fms-like tyrosine kinase 1 (sFlt-1) and soluble Endoglin (sENG) which sequester circulating vascular endothelial growth factor (VEGF) and placental growth factor (PIGF), reducing the free concentrations of these hormones in maternal plasma, and transforming growth factor β (TGFβ), as well as reducing the secretion of PIGF from the placenta (Powe *et al*. 2011, Burton *et al*. 2017). The direct cause of FGR is a failure of adequate nutrient supply via the placenta, arising from poor blood flow to and from the placenta and/or defects in exchange processes due to reduced surface area of the syncytiotrophoblast or of reduced activity of nutrient transporter proteins in this transporting epithelium (Desforges & Sibley 2010). These structural and functional changes following abnormal spiral artery conversion and remodelling provide potential targets for treating placental dysfunction (Fig. 1).

Although the origins of pre-eclampsia and FGR reside in abnormal placental development in early pregnancy, onset of both conditions can be in early or later gestation. Onset in the third trimester may be a delayed consequence of the failures of placental development in early pregnancy, for example, a failure of the capacity of a maldeveloped placenta to supply nutrients to the rapidly growing fetus in mid-to-late pregnancy, or of a separate insult such as an infection. Recent data suggest that placental inflammation occurring in late pregnancy, with either infectious or non-infectious origins, could lead to placental dysfunction (Nadeau-Vallée *et al*. 2016, Brien *et al*. 2017).

Reviewing this knowledge of the pathophysiology of pre-eclampsia and FGR suggests several possible therapeutic targets for the treatment of placental dysfunction (Fig. 1). Obviously, the most effective target would be the EVT and improving invasion into the spiral arteries where this appears to be defective. However, it is impossible at present to detect a failure in this process, occurring as it does right at the start of pregnancy, and the secondary pathologies are more amenable to treatment: improving uteroplacental and fetoplacental blood flow, combating oxidative and nitrative stress, reducing placental inflammation, reducing placental production of sFlt-1 and sEng from the pre-eclamptic placenta and stimulating placental and fetal growth. Recent work on such potential treatments is reviewed below. However, it is important first to consider the most appropriate pre-clinical strategies for testing potential treatments.

## Pre-clinical strategies for testing potential therapeutic treatments of placental dysfunction

Bearing in mind the burden of disease caused by pre-eclampsia and FGR, it may be reasonably asked why there have been so few advances in therapeutic options available to the obstetrician over the last 30 years or so and why there are so few clinical trials in this area (Fisk & Atun 2008, Chappell & David 2016). The main reason is probably the difficulty of developing drugs for conditions where there are two people involved, mother and fetus, and the consequent increased risk of side effects. This is epitomized by the thalidomide tragedy of 50–60 years ago; this drug, given as a treatment for women with hyperemesis in early pregnancy, had massive teratogenic side effects (Franks *et al*. 2004). This undoubtedly made the pharmaceutical industry even less likely to invest the large sums of money required in developing treatments for pregnancy complications. However, a secondary reason has been the absence of really well-defined strategies for good pre-clinical testing of drugs for pre-eclampsia and FGR. Broadly, three options are available: animal models, use of human placental tissue *in vitro* and mathematical modelling.

Animal models of pre-eclampsia have been recently reviewed in McCarthy *et al.* (2011), Erlandsson *et al.* (2016) and those of FGR by Swanson & David (2015). These animal models range in size from mice to sheep and pigs, and include animals spontaneously showing disease symptoms, and those arising from surgical, nutritional, pharmacological and genetic manipulations to induce pre-eclampsia and/or FGR. All have advantages and disadvantages: for example, the mouse has a placenta which is haemotrichorial (three trophoblast layers, one directly bathed in maternal blood) and so similar, though not identical, to the haemomonochorial human placenta (one trophoblast layer), can be genetically manipulated relatively easily (Dilworth & Sibley 2013) and is fairly low cost. The guinea pig is more expensive but has a haemomonochorial placenta very similar to human; the larger size of the sheep makes surgical intervention and direct access to both sides of the placenta quite straightforward, but its placenta is very different to the human, being epitheliochorial with maternal endothelium as well as trophoblast in the barrier (Swanson & David 2015).

The ideal animal model of pre-eclampsia would have similar pathology to the defective invasion by EVT of the spiral arteries found in women and would have the symptoms of pregnancy-induced maternal hypertension, renal and endothelial dysfunction and excess circulating sFlt-1 and sEng. Whilst several models show some or all of the latter symptoms, there have been virtually no reports of models with defective invasion. This is because of the major differences in placentation between species, with few showing the deep invasion by EVT found in women. Even when there are maternal symptoms, a common drawback is that these symptoms are not pregnancy specific, such as the hypertension found in the endothelial nitric oxide (eNOS) knockout mouse (Huang *et al*. 1995, Hefler *et al*. 2001, Kusinski *et al*. 2012). A very valuable exception may be the Storkhead box 1 (STOX1) transgenic mouse (Doridot *et al*. 2013). STOX1 is a transcription factor associated with human pre-eclampsia, and its overexpression in a choriocarcinoma cell lines leads to similar transcriptional changes as found in the human disease. The STOX1 transgenic mouse shows gestational hypertension, proteinuria and raised levels of sFlt-1 and sENG; it is therefore probably the most complete animal model of human pre-eclampsia described to date.

The ideal animal model of FGR would also have defective invasion of maternal arteries, and signs of a dysfunctional placenta being followed in gestation by true FGR i.e. not just a normally small fetus; there may also be maternal hypertension mimicking the pre-eclampsia with FGR found in women. In practice, no current models show the primary defect of abnormal invasion, though several show the symptoms. In our laboratory, we have found genetic mouse models of FGR to be particularly useful for testing potential therapies, for the reasons mentioned earlier (relatively cheap, similar though not identical placenta to human) and in particular the comparable cardiovascular adaptations to pregnancy and placental physiology in mice and women (Dilworth & Sibley 2013). Furthermore, the short gestation (19 days) of this species allows statistically appropriate numbers of animals to be studied quite rapidly. Development of mouse fetal weight distribution charts has helped us define FGR similarly to obstetric diagnoses (Dilworth *et al*. 2011).

We have found parallel use of the eNOS^−/−^ and placental-specific insulin-like growth factor 2 (Igf2) knockout mice to be particularly valuable in assessing therapies for FGR. The former have fetuses with an average 10% lower weight at term compared to wild-type (WT) mice with 32% falling below the 5th centile of WT weights (Kusinski *et al*. 2012). eNOS^−/−^ mouse uterine arteries have dysfunctional responses to agonists *in vitro* and the placentas show reduced activity of the System A amino acid transporter, both symptomatic of the placental phenotype in human FGR (Sibley *et al*. 2005, Kusinski *et al*. 2012). Furthermore, spiral artery remodelling and fetoplacental vascular development are impaired in eNOS^−/−^ vs WT mice, in common with human FGR (Kulandavelu *et al*. 2012,^ 2013^). The latter is associated with reduced expression of VEGF, a key promoter of placental vasculogenesis/angiogenesis (Kulandavelu *et al*. 2013).

The placental-specific Igf2 knockout (P0) mouse fetus has a reduction in weight of 20–25% compared to its WT littermates with 95% falling below the 5th centile of WT weights (Constância *et al*. 2002, Dilworth *et al*. 2010). P0 placental weight is reduced compared to WT from about embryonic day 14 (E14) onwards and so precedes the onset of FGR (Constância *et al*. 2002,^ 2005^). System A amino acid transporter activity in P0 placentas first increases vs WT at E16, probably as an adaptive response to the lower placental weight, but then decreases close to term (Constância *et al*. 2002,^ 2005^), so that the overall capacity of the small P0 placenta to transport System A amino acid substrates is reduced. The morphology of the P0 placenta is also abnormal: stereology shows that the surface area of the trophoblast layers is lower compared to WT and the thickness is increased, in common with the human FGR placenta, resulting in a reduction in the diffusional permeability (Sibley *et al*. 2004). However, uterine arteries from the P0 mouse show normal reactivity when challenged with agonists, unlike the eNOS^−/−^ mouse (Kusinski *et al*. 2011). Therefore, these two mouse models of FGR have differences in placental phenotype, which might relate to subgroups of the disease in women, and allow us to test both the general efficacy of particular drugs and obtain information on whether they are primarily acting on vascular or non-vascular targets or both (Dilworth *et al*. 2013).

One of the boons of studying the human placenta is the availability, with appropriate ethical consent, of large amounts of the organ which can be studied *in vitro*. Three different *in vitro* model systems have been developed, with increasing cellular complexity, for studies focused on the trophoblast. First, cytotrophoblast cells, the progenitor cells of the syncytiotrophoblast, can be isolated from the human placenta, delivered at any point in gestation (Kliman *et al*. 1986). These cytotrophoblast cells differentiate over the first 48 h in culture and multinucleate to form syncytiotrophoblast-like islands (Kliman *et al*. 1986). They can therefore be used to study specific effects of therapies on the syncytiotrophoblast. Secondly, placental explants are small (3 mm^3^) fragments of tissue dissected from the villous tree. These can be studied in either short-term or long-term (7 days or so) culture and have the advantage of retaining the normal architecture of the placental exchange barrier with all the cell types normally present (Simán *et al*. 2001, Greenwood & Sibley 2006). Finally, intact cotyledons, or segments, of the placenta, delivered at term can be perfused with physiological solution through the maternal and fetal circulations (Schneider *et al*. 1972, Brownbill *et al*. 1995). This is therefore the model that most similarly replicates the *in vivo* placenta with architecture intact and circulatory flow. All of these systems can be used to study placentas from both normal pregnancies and those with dysfunction associated with pre-eclampsia and FGR. Together, these three preparations are excellent for studying modes of action of particular therapies, whether they work on both normal and abnormal placentas, for studying toxicity in the most appropriate human tissue and, in the case of the perfused cotyledon, investigating pharmacokinetics of maternofetal drug transfer. However, they of course cannot provide any information of effects on the ultimate clinical systemic endpoints such as hypertension or fetal growth; only animal models can at present provide such essential pre-clinical information.

Although these trophoblast focused on *in vitro* systems, it is also possible to use isolated myometrial and chorionic plate arteries, dissected from myometrial biopsies taken at the time of Caesarean section or from the delivered placenta, respectively, from women having normal or complicated pregnancies, for studies on vascular reactivity (Wareing *et al*. 2002,^ 2004^). Wire/pressure myography allows the tension generated by the change in diameter of these arteries to be measured in the presence and absence of any drug or agent of interest. Human umbilical vein endothelial cells (HUVECs) are relatively easily isolated, are commercially available and are widely used as a model of systemic endothelial cells; microvascular endothelial cells may also be isolated from the placenta (Jones *et al*. 2015). Tong and coworkers (see references in Table 1) reported a useful strategy in a series of papers investigating drugs that might reduce sFlt-1 and sENG production, and so be useful in pre-eclampsia, using a combination of isolated primary cytotrophoblast cells, placental explants and HUVECs. This combination allowed effects on the primary endpoint (sFlt-1 and sENG secretion) to be measured and mechanisms of action to be examined, as well as determining, using the HUVECs as a model of endothelial cells, whether dysfunctionality as found in pre-eclampsia could be corrected.

**Table 1:** Summary of substances tested as potential treatments for placental dysfunction in pre-clinical studies.

Treatment	**Putative mode of action**	**Test system** (s)	**Dosing regime**	**Main outcomes of treatment** (compared to appropriate control)	Reference
Sildenafil citrate	Improved uteroplacental blood flow	See text	See text	See text	See text
Adenovirus vector with VEGF	Improved uteroplacental blood flow	See text	See text	See text	See text
Pomegranate juice (PJ)	Antioxidant	Women having normal pregnancy/placental explants and primary cytotrophoblast cells (cytos)	Women drank 8 oz/day PJ from 35 to 38 weeks gestational age (ga) to term. Explants 1% PJ in medium	Decreased placental oxidative stress *in vivo* and *in vitro*/decreased apoptosis/punicalagin key component of PJ	Chen *et al*. (2012,^ 2013^)
Tempol (superoxide dismutase mimetic)	Antioxidant	BPH/5 mouse model of pre-eclampsia	1 mmol/L from 2 days before until end pregnancy	ROS levels reduced/reduced blood pressure/reduced proteinuria/fetal and placental weights restored towards normal	Hoffmann *et al*. (2008)
		eNOS^−^/^−^ mouse model of FGR	1 mmol/L 12.5–18.5 days ga	Fetal weight increased towards normal/no effect on placental weight/UtA end diastolic velocity restored to normal	Stanley *et al*. (2012*a*)
Resveratrol	Antioxidant/enhanced NO bioavailability	COMT^−^/^−^ and eNOS^−^/^−^ mouse models of FGR	4 g/kg in diet 0.5–15.5 days ga	Fetal weight increased towards normal in COMT^−^/^−^ mouse only/no effect on placental weight/UtA minimum and maximum velocity improved towards normal in COMT mouse only/no effects on UmA velocity	Poudel *et al*. (2013)
Melatonin	Antioxidant/melatonin receptor	Lipopolysaccharide induced fetal death and FGR in mice	4 mg/kg orally in diet throughout pregnancy	Reduced fetal deaths and increased fetal weight towards normal/oxidative stress reduced	Chen *et al*. (2006)
		Ischaemia/reperfusion damage to placenta in rats	20 µg/mL orally over course of I/R experiment only	Fetal weight restored towards normal/no effect on the reduced placental weight/improved placental mitochondrial respiratory control index/decreased placental oxidative stress	Nagai *et al*. (2008)
		Undernutrition induced FGR in rats	5 µg/mL in drinking water 15–20 days ga	No effect on fetal weight but placental weight reduced so that fetal:placental weight ratio restored towards control values/restored birth weight towards control following normal delivery/upregulation of placental antioxidant enzymes	Richter *et al*. (2009)
		Nutrient restriction induced FGR in sheep	5 mg supplement daily in diet 50 days – end of experiment	Data not easy to interpret: UmA blood flow increased irrespective of nutrient intake/no effect on UtA blood flow/fetal weight only increased when nutrition adequate	Lemley *et al*. (2012)
		Nutrient restriction induced FGR in sheep	As above	Increased fetal uptake of branch chained amino acids in maternal nutrient restriction	Lemley *et al*. (2013)
		Hypobaric hypoxia (high altitude) induced FGR in sheep	10 mg/kg/day orally 100–150 days ga	Fetal weight and size further reduced/gestation increased/maternal antioxidant capacity increased	González-Candia *et al*. (2016)
Sofalcone	Hemoxygenase-1 (HO-1) induction; antioxidant enzyme	*In vitro* study: Cytos and human umbilical vein endothelial cells (HUVECs)	10, 20, 50 µmol/L in culture medium	HO-1 induced in cytos and HUVECs/decreased sFlt-1 production from cytos/suppressed endothelial dysfunction in HUVECs	Onda *et al*. (2015)
Proton pump inhibitors	HO-1 induction/anti-oxidant/anti-inflammatory/vasodilation	*In vitro* study: cytos, HUVECS and placental explants from women with severe early-onset pre-eclampsia. *In vivo* studies: sFLT-1 overexpressing and eNOS^−^/^−^ mouse models of pre-eclampsia/hypertension	*In vitro:* 5–100 µmol/L lansoprazole, rabeprazole, esomerprazole in culture medium over 24 h. *In vivo*: 150 µg esomeprazole sodium i.p. daily	sFLT-1 and sENG secretion from cytos, explants and HUVECs reduced/endothelial dysfunction reversed/vasodilated maternal blood vessels and decreased BP in mouse models/increased antioxidant protein expression/decreased secretion of cytokines	Onda *et al*. (2017)
Statins	Inhibition of 3-hydroxy-3-methylglutaryl-coenzyme A reductase (HMG-CoA) cholesterol synthesis pathway	Placental explants from early pregnancy	Cerivastatin 50 nmol/L, pravastatin 250 nmol/L in culture medium over 24 h	Proliferative effect of IGF-I and IGF-II on cytos prevented by both statins	Forbes *et al*. (2008,^ 2015^)
		Mouse model of pre-eclampsia generated by lentivral vector-mediated overexpression of sFLT-1 in placenta	Pravastatin 5 µg/day i.p. from 7.5 day ga onwards	Blood pressure lowered/proteinuria ameliorated/sFLT-1 decreased/placental growth factor (PLGF) increased	Kumasawa *et al*. (2011)
		Mouse model of pre-eclampsia generated by injection of adenovirus carrying sFLT-1 (not placenta specific)	Pravastatin 5 mg/kg/day from 9 day ga	Placental PLGF and VEGF upregulated/markers of hypoxia downregulated	Saad *et al*. (2014)
		*In vitro* study: cytos, HUVECS and placental explants from women with severe early-onset pre-eclampsia. Clinical study: 4 women with pre-eclampsia at 23–30 weeks ga	*In vitro* study: pravastatin in 20, 200, 2000 µmol/L culture medium. Clinical study: women received 40 mg pravastatin daily	sFLT-1 secretion from all *in vitro* tissues reduced/increased sENG production from HUVECs/effect on sFLT-1 mediated via HMG-CoA pathway/clinical study showed data consistent with disease stabilization	Brownfoot *et al*. (2015*a*)
		*In vitro* study similar to Brownfoot *et al*. above	*In vitro* study: comparison of simvastatin, rosuvastatin and pravastatin at 0–2000 µmol/L in culture medium	Simvastatin most potent inhibitor of sFLT-1 from all cells/all increased sENG secretion/only simvastatin upregulated HO-1 expression by placental explants from pre-eclampsia	Brownfoot *et al*. (2016*b*)
		Perfused placental cotyledons and explants (21% and 1% O_2_) *in vitro*	0.2 µmol/L pravastatin (twice the serum concentration of a 40 mg daily dose’)	No effects on sFLT-1 or PIGF secretion, or fetal perfusion pressure in perfused cotyledons/increased sFLT-1 secretion by explants under hypoxic conditions	Balan *et al*. (2017)
		11β-Hydroxysteroid dehydrogenase type 2 (11β-HSD2) knockout mouse model of FGR	20 µg/kg pravastatin i.p. daily from 6 day ga onwards	Fetal weight and placental weight increased/UmV blood velocity measurements normalized	Wyrwoll *et al*. (2016)
C-1	Nitric oxide induction, guanylyl cyclase activation/HIF1α inhibition	*In vitro* study: cytos, HUVECS and placental explants from women with severe early-onset pre-eclampsia	0–100 µmol/L in culture medium 24–72 h	sFLT-1 and sENG secretion from cytos, explants and HUVECs reduced/endothelial dysfunction reversed/HIF1α expression by explants reduced	Brownfoot *et al*. (2015*b*)
Metformin	HIF1α inhibition via blocking of mitochondrial electron transport chain inhibition	*In vitro* study: cytos, HUVECS and placental explants from women with severe early-onset pre-eclampsia	0–1 mmol/L in culture medium 24–72 h	Similar results to YC-1: sFLT-1 and sENG secretion from cytos, explants & HUVECs reduced/endothelial dysfunction reversed/HIF1α expression by explants reduced. Evidence that effect via mitochondrial electron transport chain complex 1	Brownfoot *et al*. (2016*a*)
[Leu27] insulin-like growth factor-II (IGF-II)	IGF-II receptor antagonist – increasing IGF-II bioavailability	eNOS^−^/^−^ mouse model of FGR	1 mg/kg/day sc 12.5–18.5 day ga	Reduction in number of FGR (<5th centile) pups	Charnock *et al*. (2016)

Mathematical, *in silico*, modelling of the human placenta is still in its infancy, but studies show that it has great promise for investigating and shedding new light on complex issues such as amino acid transfer across the placenta and determinants of blood flow in the intervillous space (Widdows *et al*. 2015, Panitchob *et al*. 2016). As these models develop, they will provide means of rapidly screening potential therapies and information for focusing *in vitro* work.

**Figure 2:**
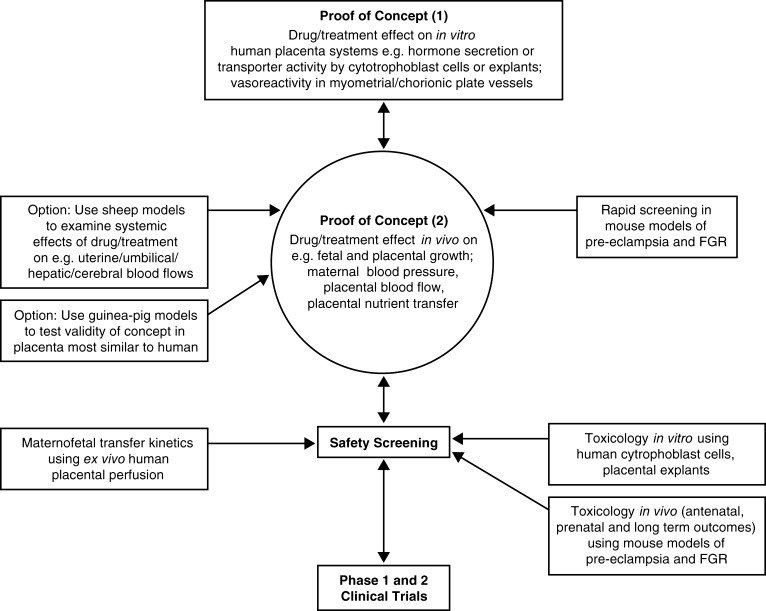
Proposed experimental pathway for taking a potential drug or treatment for placental dysfunction from concept through to early phase clinical trials.

In summary, whilst there is no single ideal pre-clinical model, there are extremely well-characterized animal and *in vitro*systems which, used in combination, are excellent tools for determining the effectiveness of potential treatments of placental dysfunction. An experimental pipeline for taking a potential drug or treatment for placental dysfunction from concept through to early phase clinical trials is shown in Fig. 2. This provides a starting point for debate on the ideal pathway. One area for discussion is on the usefulness of non-human primates in these studies. They are not included in Fig. 2 because of their expense, the technical challenges involved in their use and the ethical sensitivity, all of which require work to be done in specialist centres. On the other hand, they could accelerate translation and possibly reduce the need for some early phase clinical trials.

## Potential treatments for placental dysfunction emerging from pre-clinical studies

For the reasons already described, there are no drugs being developed by the pharmaceutical industry to specifically treat placental dysfunction. Therefore, the broad strategy recently used by a number of groups working in this area is to identify drugs: (1) that have been developed to treat other diseases but which target pathways that could be useful in placental dysfunction e.g. vasodilators, oxygen/nitrogen free radical scavengers; (2) for which there is at least some pre-existing evidence for safety in pregnancy. Identified drugs have then been tested using a mix of the pre-clinical models reviewed above. Sildenafil citrate provides a good example of where this kind of strategy has been applied with contributions from a number of different laboratories over a number of years. Adenovirus-mediated delivery of VEGF provides a good example of a somewhat different strategy with a more systematic approach being taken by a single group of researchers to the pre-clinical development of a treatment to increase uteroplacental blood flow. These two examples are considered in more detail below, whilst Table 1 provides a summary of substances that have been tested so far as potential direct treatments of placental dysfunction.

### Sildenafil citrate (Viagra) as a treatment for placental dysfunction

Sildenafil citrate (SC) is a phopshodiesterase-5 inhibitor which blocks the breakdown of cGMP and so enhances nitric oxide (NO)-mediated vasodilation. Therefore, although designed as a treatment for pulmonary hypertension and later used successfully to treat erectile dysfunction in men, SC could be useful to improve perfusion through the uteroplacental circulation. A systematic review of a small number of clinical and animal pregnancy studies in 2007 suggested that SC had no teratogenic or other adverse effects in animals or women (Villanueva-García *et al*. 2007) and so the drug fits the criteria for a potential treatment of FGR. With this in mind, Wareing and coworkers (Wareing *et al*. 2005) investigated whether SC would improve vasodilation in small myometrial arteries mounted in the wire myograph. They found that vessels from FGR placentas showed increased artery vasoconstriction and decreased endothelial-dependent vasodilation as compared to those from normal placentas and that both these effects were reversed by SC. This encouraged further pre-clinical studies of the drug.

Disappointingly, and of concern, a sheep model of FGR produced by single umbilical artery ligation (Miller *et al*. 2009) found that SC administered as a 100 mg bolus after 24 -h infusion (4.17 mg/h) at around day 115 of gestation (term in sheep is 150 days) had no effect on uterine blood flow, and in fact increased fetal heart rate. The reasons for these unexpected effects of SC were not clear. A negative effect of SC on pup weight was also found in a rat model where FGR was induced by administration of Nω-nitro-l-arginine methyl ester (l-NAME), a blocker of nitric oxide synthase, by gavage from days 14 to 19 of gestation (term in the rat is days 21); SC administration via the same route actually reduced pup weight greater than l-NAME alone (Nassar *et al*. 2012). Clearly gavage is not a clinically relevant route of administration.

By contrast, in another sheep study, this time using nutrient restriction to induce FGR, Satterfield and coworkers (Satterfield *et al*. 2010) found that SC, administered as a daily subcutaneous injection from 28 to 115 days gestation at a maximum of 150 mg/day, improved both fetal weight and amino acid availability; no blood flow measurements were reported. The disparity between the two sheep studies obviously could be related to either the method used to induce FGR or the very different SC dosing regimes. Most recently, in a third different sheep model in which uterine artery embolization between days 102 and 107 of gestation was used to induce FGR, SC was administered, at the same dose as that used by Satterfield and coworkers, via subcutaneous infusion from the completion of embolization to when pregnancy was terminated at days 132 and 133 of gestation (Oyston *et al*. 2016). Lamb and placental weights were reduced in the embolized group as compared to control but not when SC was administered: the recovery towards control values with SC was most noticeable in regard to placental weight. Umbilical artery Doppler measurements showed that whilst Resistive Index in the embolized group fell less than controls over gestation, values in the SC-treated group were intermediate between the two. The authors speculate that changes in placental growth with SC were more important than changes in placental efficiency.

In a nutrient restriction rabbit model of FGR (López-Tello *et al*. 2016), SC administered orally from days 22 to term (days 31) returned crown rump length and biparietal diameter to control (normal nutrition) size, though fetal weight was still as low as in the restricted group. Whilst placental weight was not different between groups, histopathology suggested that SC improved placental vascularity. Interestingly, SC also increased liver to body weight ratio above than that in either nutrient-restricted or control groups. Umbilical artery Doppler measurements in this study showed that nutrient restriction only had an effect on systolic peak velocity, and this variable was similarly above control values in the SC group. However, Doppler measurement of middle cerebral artery indices showed that SC increased resistance index and pulsatility index above the values in the control and nutrition-restricted groups; the authors suggest that this effect of SC could counteract excessive blood flow to the brain and so prevent cerebral oedema.

We found that SC improved fetal growth in two different genetic models of FGR (Stanley *et al*. 2012*b*, Dilworth *et al*. 2013). The catechol-O-methyltransferase knockout (COMT^−^/^−^) mouse was previously reported to have a similar phenotype to pre-eclampsia including hypertension in pregnancy, proteinuria and FGR (Kanasaki *et al*. 2008). In our study, whilst the COMT^−^/^−^ mouse had a higher systolic pressure than wild-type (WT) mice prior to pregnancy and at day 10, there was no difference later in gestation. Proteinuria (measured as creatinine:albumin ratio) was genotype dependent. Neither variable was affected by SC (administered in drinking water at 0.2 mg/mL from day 12.5 onwards). However, pup weight at the end of pregnancy was significantly lower in the COMT^−^/^−^ mouse than in WT mice, and SC partially reversed this effect. Placental weight was higher in the knockout than in WT, and this difference was unaffected by SC. Uterine artery Doppler measurements were not different between COMT^−^/^−^ and WT and were unaffected by SC. Interestingly, uterine arteries from the COMT^−^/^−^ mice studied *in vitro* in the wire myograph did show increased constriction to phenylephrine, as compared to those from WT mice, and this was normalized by SC: these data are similar to those reported by Wareing *et al.* (2005) for the human myometrial vessels. Umbilical artery Doppler measurements, pulsatility index and resistance index were higher in the COMT^−^/^−^ vs WT mice and were normalized by SC. These data suggest that the COMT^−^/^−^ mouse as used here is a better model of FGR than of pre-eclampsia, and that SC is effective in reversing FGR by improving fetoplacental vascular function.

As described above, the P0 mouse provides a model of FGR phenotype with normal placental vascular function and exchange barrier dysfunction. Bearing in mind the assumption, and indeed the evidence discussed here, that SC improves fetal weight in FGR models through an effect on blood flow, we tested the drug in P0 mice with the hypothesis that it would have no effect on fetal growth (Dilworth *et al*. 2013). Contrary to this, we found that SC, administered similarly to the COMT^−^/^−^ study, did improve fetal weight in P0 fetuses, as compared to untreated P0 animals: 49% of P0 fetuses were above the 5th centile of untreated WT weights as compared with 25% in the untreated P0 group. Placental weight was lower in the untreated P0 vs WT but was increased by SC in the former but not latter. Umbilical artery and vein blood flow velocity measurements were not different between P0 and WT as expected and were unaffected by SC. Measurements of unidirectional methylaminoisobutyric acid (a non-metabolizable amino acid analogue and substrate of System A) clearance across the placenta suggested that SC increased fetal weight in P0 animals by improving total placental transfer capacity (combination of small effects on placental size and transporter activity).

The majority of these pre-clinical studies therefore support the effectiveness of SC in treating placental dysfunction in FGR. The dosing regime has been an important factor in effectiveness in *in vivo* studies, and SC’s mode of action seems to be more complex than a simple effect on placental perfusion with effects on placental growth, vascularity and transfer capacity also evident.

Building on the pre-clinical studies SC has been used in clinical case studies (Panda *et al*. 2014, Choudhary *et al*. 2016, Sakamoto *et al*. 2016) and a small case-control trial (von Dadelszen *et al*. 2011) all of which show that SC can improve outcome of FGR in women. Two prospective randomized trials (Trapani *et al*. 2016, El-Sayed *et al*. 2017) have now shown that sildenafil can improve uterine and umbilical artery Doppler indices in FGR suggesting that it does improve uteroplacental blood flow in such pregnancies. These studies all provide the foundation for the ‘sildenafil therapy in dismal prognosis early-onset intrauterine growth restriction’ (STRIDER) trial, a double-blind prospective trial of SC which will report in 2017 (Ganzevoort *et al*. 2014). By providing definitive data on the potential of SC for treating FGR, this trial will also help to determine the credibility of the pre-clinical strategy for discovering treatments of placental dysfunction as described above.

### Adenovirus-mediated delivery of vascular endothelial growth factor

David and coworkers have taken a different, rather more sophisticated approach to increasing uteroplacental blood flow in FGR and pre-eclampsia. They hypothesized that overexpression of VEGF in uterine arteries using an adenovirus vector system would lead to increased uteroplacental blood flow and therefore fetal growth (David *et al*. 2008). This hypothesis has been tested in a series of pre-clinical studies using sheep and guinea pig models of FGR as well as *in vitro* studies using the human placenta. First, adenovirus vectors encoding the VEGF-A gene (Ad.VEGF) were injected into the uterine arteries of sheep having normal pregnancies; the data showed that the Ad.VEGF caused a sustained increased in uteroplacental blood flow, whereas vector containing the marker gene beta-galactosidase (Ad.LacZ) had no effect (David *et al*. 2008, Mehta *et al*. 2012). Mehta *et al*. (2012) also reported data suggesting that short-term effects of Ad.VEGF were via upregulation of eNOS, whereas longer-term effects most likely involved increased uterine artery vascularization. They also showed that the human VEGF had not crossed the placenta and that there were no obvious undesirable effects of the adenovirus on mother or fetus. Next, in a sheep model of FGR where overnutrition of adolescent ewes causes reduced uteroplacental blood flow and decreased lamb birth weight near term, Ad.VEGF injection into the uterine artery resulted in improved lamb abdominal circumference, but no significant difference in fetal weight when compared to suitable controls was observed (Carr *et al*. 2014). However, Ad.VEGF injection in the overnutrition group did lead to fewer lambs with birth weights greater than 2 s.d. below the non-FGR control mean; this is an important finding as clinically small improvements in fetal growth, with subsequent delay in time of delivery, could improve survival and morbidity rates in human FGR. Surprisingly, the authors could not find any effects of Ad.VEGF on uteroplacental blood flow in this study, perhaps due to technical limitations of their measurements. Interestingly, uteroplacental arteries studied in the myograph *in vitro* did show improved vasodilation and relaxation. However, the study also showed that treatment with Ad.VEGF resulted in upregulation of placental VEGF receptors, suggesting (as with sildenafil as described above) a potentially different, additional, mode of action than that originally hypothesized. More recently (Carr *et al*. 2016), using the same sheep model of FGR again showed that Ad.VEGF administration increased abdominal circumference at mid gestation as compared to saline-treated controls with a trend towards higher birth weight at term; there were no untoward postnatal effects of the treatment.

Moving to guinea pigs as a more appropriate model of human placentation than sheep, the optimal method for gene delivery to the uterine arteries in guinea pigs was investigated (Mehta *et al*. 2016). Delivery of adenovirus containing the gene for beta-galactosidase was accomplished by intravascular injection into the uterine artery or into internal iliac arteries, by external administration whereby vector in PBS was dribbled onto the uterine artery or by external vascular administration of the vector in pluronic gel, a substance that is liquid at room temperature but which becomes a gel at body temperature. The use of pluronic gel was the most successful in every way: success rate of administration, maternal and fetal survival and transduction efficiency with no spread to maternal or fetal tissues. Using this method of administration, Swanson *et al.* (2016) investigated whether Ad.VEGF would improve fetal growth in an undernutrition model of guinea pig FGR, comparing to similar animals treated with adenovirus encoding a reporter gene, beta-galactosidase (Ad.LacZ). They found a very small (3%) higher fetal weight in the Ad.VEGF-treated pups at term (60–64 days) with no effect on litter size. There was no effect on placental weight, although placental depth was increased; the weights of fetal liver, brain and lung were increased. VEGF expression was clearly higher in uterine arteries treated with Ad.VEGF than those treated with Ad.LacZ, and ELISA measurements showed that VEGF levels in fetal serum were also higher in the former group, though there were no differences in maternal serum. Importantly, using RT-PCR, adenovirus was only found in the vessels where it had been applied and not in any other maternal or fetal tissue. Uterine arteries, studied in the myograph, constricted similarly when exposed to acetylcholine whether they had been exposed to Ad.VEGF or Ad.LacZ *in vitro*, but there was a clear difference in the two sets of vessels in terms of acetylcholine-induced vasorelaxation, with Ad.VEGF exposed vessels showing significantly greater relaxation. This may have been related to increased iNOS and eNOS expression in these vessels. There was also evidence that Ad.VEGF increased vascularization in the vessel adventitia, similar to that found in the sheep work, suggesting a longer-term mechanism for its mode of action. Overall, although the very small effect on fetal growth is a concern, this study in an animal model resembling the human shows that Ad.VEGF is a viable treatment for FGR and provides a significant step forward from the sheep work in translating this strategy to the clinic.

Translation of Ad.VEGF therapy to the clinic has been supported by work on the human placenta *in vitro* to determine potential adverse effects (Brownbill *et al*. 2014). Ad.VEGF-D at a range of doses had no effect on villous explant integrity and function and similarly had no deleterious effects when perfused through the maternal side of placental cotyledons. There was minimal virus vector tissue uptake, and the vector was rarely detected in fetal venous perfusate, and then only at low titre, suggesting little transplacental transfer.

This combination of large and small animal work together with toxicity testing on the human placenta *in vitro* has underpinned a Europe wide phase 1/2a clinical trial investigating the efficacy and safety of adenoviral VEGF gene therapy as a treatment for severe early-onset FRG – the EVERREST Trial – which is now in development (Sheppard *et al*. 2016).

### Other substances tested as potential treatments for placental dysfunction in pre-clinical studies

Table 1 summarizes a range of studies on a number of different potential treatments for placental dysfunction. As can be seen, these have used the wide variety of *in vivo* and *in vitro* models described above. Whilst, as already noted, these provide a range of useful data, the diversity of models, the different dosing regimes and different times in gestation when they are applied do not provide the best foundation for translation into early phase clinical trials. Now a number of different groups are working towards treatments for placental dysfunction, and it is clear that an agreed optimal framework for such work would be helpful and indeed essential for reducing the numbers of animals being used and the costs for delivering treatments to the clinic.

As also can be seen from Table 1, a number of different antioxidants have been considered. Of these, melatonin has received the most attention: several, but not all, studies have shown a positive effect on fetal weight in FGR, though in one sheep study melatonin actually worsened the FGR (González-Candia *et al*. 2016). Nevertheless, melatonin has been tested in clinical trials as a treatment for both pre-eclampsia and FGR (Alers *et al*. 2013, Hobson *et al*. 2013): the outcomes of these trials are awaited.

Statins have also received considerable attention. These inhibitors of HMG-CoA reductase in the cholesterol synthesis pathway have reduced sFlt-1 and sENG secretion by the placenta *in vitro* and *in vivo* in a number of studies, though there was no effect on sFlt-1 production in one human placental perfusion study (Table 1). Also of note is *in vitro* work suggesting that statins can prevent IGF-I and II stimulation of cytotrophoblast cell proliferation, with a potential harmful effect on placental growth (Forbes *et al*. 2008). However, Costantine *et al.* (2016) have recently reported a small safety and pharmacokinetic trial of pravastatin for the treatment of pre-eclampsia (10 women receiving pravastatin, 10 receiving placebo) and found no adverse effects on mother or baby. Although this was a secondary outcome, pre-eclampsia was reported in 4 of the women receiving placebo but none in those receiving pravastatin; birth weight was similar in the two groups, but placental weight was not reported.

A number of other potential treatments against different placental therapeutic targets are shown in Table 1 with positive results. It is to be hoped that these will stimulate the further work required before clinical trials can be considered. The largely positive reports in Table 1 do raise the possibility that other substances have been tested but, with no effect, the data have not been reported in the published literature. It is particularly important that in a small field like placentology that a forum for the reporting of such negative data is found so as to prevent unnecessary duplication and highlight modes of action not worth pursuing.

## Opportunities and challenges

The work described in this review shows that it is now possible to develop treatments targeted to placental dysfunction. Useful *in vitro* and *in vivo* test systems are available and the strategy of repurposing drugs developed for other diseases does provide a pipeline for the placentologist to work with. The outcomes of trials such as STRIDER and EVERREST are eagerly awaited to determine whether this strategy works all the way into the clinic. The challenges cannot be underestimated of course. The need for an agreed framework to pre-clinical studies in this field and for a means of reporting negative results has already been mentioned. The minimization of risk to both mother and fetus of treatments for placental dysfunction has to be the priority; post-natal and later life risks of these treatments must be considered very carefully, both in pre-clinical work and in early phase clinical trials. The work on targeting therapies using adenoviral vectors is exciting both in terms of improving efficacy of treatments and reducing side effects. Also very promising in this regard is the use of nanoparticles to selectively deliver drug payloads. King and coworkers (King *et al*. 2016) recently reported that such nanoparticles, decorated with placenta specific homing peptides, can selectively deliver IGF-II to the mouse placenta and, in the P0 mouse model of FGR, improve fetal weight. Targeting drugs to the placenta in this way will hopefully reduce risk and perhaps persuade pharma that it is possible to get involved in developing new drugs with which to treat placental dysfunction.

It is clear from the pathophysiology of placental dysfunction, and indeed from clinical observation that the disease has different phenotypes in different women (Sibley *et al*. 2005). For example, uteroplacental blood flow reductions may be paramount in some cases of FGR, whereas placental trophoblast dysfunction, with abnormalities in hormone secretion and in maternofetal transport, may be the prime defect in other cases. Such stratification of placental disease, with consequent development of phenotype specific drugs, coupled to selective methods of delivery, such as those described above, will transform the treatment of the dysfunctional placenta.

## Footnote

This article is adapted from work presented at the Aspen/Snowmass Perinatal Biology Symposium, 27–30 August 2016. The meeting was supported in part by the *Journal of Endocrinology*.

## Declaration of interest

The author declares that there is no conflict of interest that could be perceived as prejudicing the impartiality of this review.

## Funding

The author is very grateful to the funders of work in his laboratory on the topic of this review, primarily, The Wellcome Trust, The Medical Research Council, Tommy’s The Baby Charity and an Action Research Endowment Fund.
